# Late-onset *epi-cblC* methylmalonic aciduria with tissue-variable *MMACHC* promoter methylation due to a stop retained *PRDX1* variant

**DOI:** 10.1186/s13148-026-02135-8

**Published:** 2026-04-19

**Authors:** Martina Škopková, Pavlína Kabelíková, Andrea Andrésová, Katarína Brennerová, Silvia Dallemule, Miroslav Sabo, Róbert Petrovič, Daniela Gašperíková

**Affiliations:** 1 Institute of Experimental Endocrinology, Biomedical Research Center, Slovak Academy of Sciences, Dúbravská cesta 9, Bratislava, 84505 Slovakia; 2 Institute of Experimental Oncology, Biomedical Research Center, Slovak Academy of Sciences, Bratislava, Slovakia; 3 Department of Paediatrics, National Institute of Children’s Diseases, Bratislava, Slovakia; 4 Institute of Medical Biology, Genetics, and Clinical Genetics, Medical Faculty, University Comenius and University Hospital Bratislava, Bratislava, Slovakia

**Keywords:** *MMACHC*, Vitamin B12 metabolism disorder, Late-onset *epi-cblC*, Stop retained variant, Aberrant splicing, Epimutation

## Abstract

Mutations in the *MMACHC* gene lead to autosomal recessive combined methylmalonic aciduria and homocystinuria (*cblC* type). Additionally, specific mutations in the downstream, inversely oriented *PRDX1* gene that result in aberrant *PRDX1* transcripts overlapping the *MMACHC* promoter lead to its methylation and consequent downregulation of expression. This type is called *epi-cblC* and can be due to bi-allelic *PRDX1* mutations causing *MMACHC* epimutation, or it can be digenic with heterozygous *PRDX1* and *MMACHC* pathogenic variants located *in trans*. We identified a patient with an unusually late-onset *epi-cblC* type due to the compound heterozygous recurrent pathogenic *MMACHC*:c.271dup, p.(Arg91Lysfs*14) variant and a novel *PRDX1*:c.599G > A, p.(Ter200=) variant. We found that the *PRDX1* stop retained variant caused activation of a cryptic acceptor splice site and production of an aberrant transcript overlapping the *MMACHC* promoter. We confirmed hemimethylation of the *MMACHC* promoter in the blood of the patient and her daughter, also a carrier of the *PRDX1* stop retained variant. Interestingly, the methylation level was much lower in the patient’s fibroblasts. In line with these methylation data, expression of the *MMACHC* gene was attenuated in blood, while it was preserved in the fibroblasts of the proband. The difference in methylation could be explained by a lower proportion of aberrant *PRDX1* transcripts detected in fibroblasts compared to blood. In conclusion, we describe a rare example of a stop retained variant causing aberrant splicing with a tissue-dependent level of expression. These differentially expressed splicing changes presumably lead to varying degrees of *MMACHC* promoter methylation and gene expression observed in blood and fibroblasts. We suggest that preserved *MMACHC* expression from one allele in a subset of tissues could explain the unusual late onset and milder form of the disease in the *epi-cblC* type patient.

**Supplementary Information:**

The online version contains supplementary material available at 10.1186/s13148-026-02135-8.

## Background

In addition to a vitamin B12 (cobalamin) deficiency, combined methylmalonic aciduria and homocystinuria can result from recessive pathogenic variants in genes involved in the common pathway of adenosyl-cobalamin and methyl-cobalamin synthesis from vitamin B12 (reviewed in [1]). Adenosyl-cobalamin serves as a cofactor for methylmalonyl-CoA mutase, whose impairment leads to accumulation of methylmalonic acid (MMA). Methylcobalamin is a cofactor of methionine synthase, which is important for methylation and re-methylation processes, and its impairment leads to the accumulation of homocysteine (Hcy). The most common cause of impaired intracellular cobalamin metabolism resulting in combined methylmalonic aciduria and homocystinuria is mutations in the *MMACHC* gene (Methylmalonic aciduria and homocystinuria, *cblC* type, MIM 609831). The MMACHC protein functions as a chaperone that facilitates cobalamin trafficking through the cytoplasm and its subsequent interaction with MMADHC in the metabolic pathway [2].

Recently, specific variants in the *PRDX1* gene have been identified as a cause of hypermethylation of the *MMACHC* promoter, leading to decreased *MMACHC* expression [3–5]. *MMACHC* and *PRDX1* belong to a group of genes organized as trios of reverse (R1)/forward (F2)/reverse (R3) genes, where *MMACHC* is a sense gene flanked upstream by *CCDC163P* and downstream by *PRDX1* in the opposite orientation. *MMACHC* and *CCD163P* are regulated by a common bidirectional promoter. It was suggested that epivariations in the R1/F2/R3 trios of genes represent a general mechanism, in which epivariation in the bidirectional promoter R1/F2 could be produced by aberrant transcription from R3, triggered by gene variants and/or environmental factors [6]. The hypermethylation of the *MMACHC* promoter observed in *cblC* patients was shown to be caused by *PRDX1* splicing mutations that give rise to aberrant *PRDX1* transcripts, which are antisense to and overlap the *MMACHC* gene [3]. This type of combined methylmalonic aciduria and homocystinuria has been termed *epi-cblC* and can be either digenic, in which a *PRDX1* mutation is combined with a *MMACHC* pathogenic variant, or due to bi-allelic *PRDX1* mutations. To date, three *PRDX1* variants c.515-1G>T, c.515-2A>T [3, 4], and c.*2C>T [5] have been reported in a total of 21 patients. The phenotype of these *epi-cblC* patients matches that of patients with classical *cblC* type [4]. As in *cblC*, most *epi-cblC* cases are with neonatal or infantile onset, and they present with intrauterine growth retardation, lethargy, failure to thrive, feeding difficulties, hypotonia, seizures, developmental delay, cognitive impairment, retinopathy, cardiac and hematologic abnormalities [4, 7, 8]. Late-onset cases are less frequent and have a more favorable outcome. The main symptoms in these cases include neurological manifestations that include walking difficulties, peripheral neuropathy, psychiatric disorders, pyramidal syndrome, anemia, and high blood pressure [9]. Among the 21 *epi-cblC* patients reported thus far, only three had the later onset. In two cases, the ages at onset were 14 [10] and 59 [3] years. In one case with a complex medical history, the clinical and biochemical diagnosis of combined methylmalonic aciduria and homocystinuria was made at the age of 63, but the first symptoms related to *epi-cblC* could have emerged in the third decade of life [4].

Here, we describe a novel *PRDX1* TGA stop to TAA stop codon retained variant leading to aberrant *PRDX1* splicing and hypermethylation of the *cis*-located *MMACHC* promoter in a patient with late-onset combined methylmalonic aciduria and homocystinuria.

## Methods

### Subjects

The index case (Proband, P), a 65-year-old female, is described in detail in the Results section. The patient’s 38-year-old healthy daughter (D) was included in the investigation. The controls included healthy women aged 40 (Control 1, C1), 55 (Control 2, C2), and 30 (Control 3, C3). Informed consent for genetic testing was obtained for all participants, and consent for a skin biopsy was obtained for the proband and healthy controls.

### Laboratory diagnostics

MMA was quantified in urine by examination of the organic acid profile using gas chromatography-mass spectrometry (GC-MS) on an ITQ 900 (Thermo Scientific). Concentration of ammonia in plasma was measured by dry chemistry using the Vitros 4600 system (Ortho Clinical Diagnostics). Total Hcy in plasma was determined using the above-mentioned biochemistry analyzers with both Ortho’s original and a commercially available test (Axis Shield Diagnostic LTD).

### Cell culture

The fibroblast cell line from the proband, as well as the control fibroblasts, were established from a skin biopsy by the explant method. The cells were cultured in DMEM medium (Gibco) supplemented with 10% FBS (Gibco) and 1% Penicillin-Streptomycin (Gibco) at 37 °C and 5% CO_2_.

### Nucleic acid extraction

Peripheral blood for DNA extraction was withdrawn into standard K2EDTA tubes (Sarstedt). Whole blood DNA was extracted using the MagCore Genomic DNA Whole Blood Kit (RBC Bioscience). Fibroblasts in adherent culture reaching 70–90% confluency were washed with 1x PBS (Gibco), detached with 1x trypsin-EDTA (Gibco), and harvested with complete DMEM medium. After centrifugation at 300 g for 5 min, the cells were resuspended in 1x PBS, and DNA was extracted using the MagCore Cultured Cells DNA Kit (RBC Bioscience). The quality and quantity of DNA were checked using a Nanodrop (ThermoFisher), and the DNA was stored at -20 °C.

Peripheral blood for RNA extraction was collected into a Tempus Blood RNA Tube (Applied Biosystems) containing a stabilizing agent, and RNA was isolated using the MagMAX for Stabilized Blood Tubes RNA Isolation Kit (Invitrogen), with a DNase step included in the protocol. RNA from the fibroblasts was extracted using the RNeasy Mini kit (Qiagen), including DNA removal with DNase I (Qiagen). Fibroblasts grown in adherent culture were washed with 1x PBS (Gibco) and lysed directly on the 6-well plate. The extracted RNA was checked for quality and quantity using a Nanodrop (ThermoFisher) spectrophotometer, and its integrity was assessed by 1% agarose gel electrophoresis stained with GelRed (Biotium). RNA was stored at -80 °C.

### Whole exome sequencing (WES)

Peripheral blood DNA was used for whole-exome sequencing performed as a service by Novogene, UK, using the Agilent SureSelect XT V6 kit for library preparation. Secondary bioinformatic analysis was performed by Novogene, as well. Briefly, reads were aligned to the GRCh37 reference genome using Novoalign version 3.02.13 (Novocraft) with default parameters. After genome alignment, the conversion from SAM format to BAM and duplicate removal were performed using Picard Tools (2.20.8). The Genome Analysis Toolkit, GATK (3.8) [10], was used for local realignment around indels, base recalibration, variant recalibration, and variant calling. Variants were annotated and prioritized using an in-house Gemini SQLite database [11]. A virtual panel of genes linked to intracellular cobalamin metabolism was analyzed (*ABCC1*,* ABCD4*,* ACSF3*,* ALDH6A1*,* AMN*,* CBS*,* CD320*,* CTRB1*,* CTRB2*,* CUBN*,* CBLIF*,* HADH*,* HCFC1*,* BLTP1*,* L2HGDH*,* LMBRD1*,* LRP2*,* MCEE*,* MLYCD*,* MMAA*,* MMAB*,* MMACHC*,* MMADHC*,* MTR*,* MTRR*,* MMUT*,* OXCT1*,* PCCA*,* PCCB*,* PRSS1*,* PRSS3*,* RBSN*,* SUCLA2*,* SUCLG1*,* TCN1*,* TCN2*).

### Sanger sequencing

All used primers are listed in the Suppl. Table 1. Primers for amplification of exon 2 of the *MMACHC* (NM_015506.3) and exon 6 of the *PRDX1* (NM_181697.3) genes were used for verification of candidate variants identified by WES and for co-segregation in the family as described previously [5]. Primers PRDX1_S and CCDC163P_AS were used for amplification and sequencing of the part of the aberrant transcript overlapping the *MMACHC* gene from cDNA (see reverse transcription) as described previously [3].

### mRNAseq

RNA extracted from peripheral blood or cultured fibroblasts was sequenced as a service at Novogene, UK. Blood RNA was globin-depleted, and mRNA was enriched using poly-T oligo-attached magnetic beads for both tissue types. The obtained FASTQ files were processed using the nf-core/refseq nextflow pipeline version 3.19.0 [12] with the STAR aligner used for mapping to the GRCh38 reference genome and projecting the alignments onto the transcriptome obtained from GENCODE v48 [13]. BAM-level quantification was done using the Salmon tool [14]. Alignments and sashimi plots were visualised by the Integrative Genomics Viewer (IGV) v.2.18.4 [15].

### Nanopore sequencing

Amplicons of the aberrant transcript generated using primers PRDX1_S and CCDC163P_AS were purified using the QIAquick PCR Purification Kit (Qiagen) and quantified with the Qubit dsDNA High Sensitivity Assay Kit (Thermofisher Scientific). Barcoded libraries were prepared using the Ligation Sequencing Kit (SQK-LSK109) with Native Barcoding Expansion (EXP-NBD104) (Oxford Nanopore Technologies). The final pooled libraries were sequenced using R9.4.1 flow cells (FLO-MIN106) on a MinION Mk1C instrument (Oxford Nanopore Technologies). Raw barcoded reads were basecalled and demultiplexed using the MinKNOW v.23.04.5 software. The resulting FASTQ files were mapped to the GRCh38 reference human genome using the Minimap2 v.2.28 tool pre-set for splice-aware alignment for long reads [16].

### Sodium bisulfite modification

Sodium bisulfite (BS) treatment of the extracted genomic DNA was performed using the EpiTect Bisulfite kit (Qiagen, Hilden, Germany) following the manufacturer’s protocol. Modified DNA aliquots were stored at − 18 °C. This treatment converts all unmethylated cytosines to uracil, leaving 5-methylcytosines unchanged.

### Methylation-specific PCR and qPCR

Primers specific for unmethylated and methylated promoter sequences after bisulfite (BS) conversion (Suppl. Table 1) were used as described by [5]. The reaction conditions were: 50 ng of BS-treated DNA was added to the reaction mix containing HotFIREPol polymerase (Solis Biodyne) in 1x B1 buffer, 200 µM dNTPs, 2.5 mM MgCl_2_, and 0.2 µM forward and reverse primers each. The cycling conditions included an initial denaturation step at 95 °C for 15 min, followed by 35 cycles of denaturation at 95 °C for 30 s, primer annealing at 52 °C for 1 min, and polymerisation at 65 °C for 30 s, and a final polymerisation step at 72 °C for 10 min. Amplicons were visualised on 2.5% agarose electrophoresis stained with GelRed (Biotium). For quantification, the same reaction mix was used with the addition of 0.1x SYBR Green I (Thermo Fisher), and the same cycling conditions were applied, except that the final polymerization step was replaced with melt curve analysis on the QuantStudio 5 instrument (Thermo Fisher). Results were analysed using the QuantStudio Design & Analysis software (Thermo Fisher). Formula 2^−ΔΔCt^ was used to assess the ratio of methylated and unmethylated target sequence, which was then converted to percentages of the methylated target. The specificity of the PCR reaction was checked using the melting curve and visualization on a 2.5% agarose gel stained with GelRed (Biotium).

### Pyrosequencing

The methylation level of the *MMACHC* promoter was measured by quantitative pyrosequencing in BS-treated blood DNA of the patient, as well as in the BS-treated DNA from the patient’s skin fibroblasts. Forward and biotinylated reverse amplification primers, as well as pyrosequencing primers for 3 regions of the *CCDC163P/MMACHC* bidirectional promoter (Suppl. Table 1), were designed using the PyroMark Assay Design software (Qiagen). The region of interest was amplified from BS-modified DNA using amplification primers and the Q PyroMark PCR kit. After purification and denaturation, the biotinylated PCR product was sequenced using the pyrosequencing primer with the PyroMark Q24 Pyrosequencing System (Qiagen). The obtained pyrosequencing data were analysed using the PyroMark Q24 2.0.6 software (Qiagen). The results are presented as the percentage of methylation at CpG sites (averaged from 2 to 6 measurements), and the values ​​were normalized to the C1 blood DNA control.

### Reverse transcription

The cDNA was prepared from 1 µg of RNA annealed with 50 pmols of random hexamers (Invitrogen) after denaturation at 65 °C for 5 min and rapid cooling on ice. The RNA was reverse-transcribed using 200 U of SuperScript III Reverse Transcriptase (Invitrogen) in the presence of 1x First Strand Buffer (Invitrogen), 0.5 mM dNTP (Solis Biodyne), 5 mM DTT (Invitrogen), and 40 U of RNaseOUT Ribonuclease inhibitor (Invitrogen). The reaction mix was incubated for 5 min at 25 °C, 60 min at 50 °C, and inactivated for 15 min at 70 °C.

### Quantitative real-time PCR

Quantitative real-time PCR (qPCR) for the *MMACHC*, *PRDX1*, and *YWHAZ* genes was done using TaqMan chemistry. One µl of cDNA was used in a 10 µl reaction volume with pre-designed FAM-labelled TaqMan assays (Hs01029779_g1, Hs00602020_mH, Hs03044281_g1, respectively, Thermo Fisher) and the TaqMan Gene Expression Master Mix (Thermo Fisher), according to the manufacturer’s instructions. The qPCR was performed on the QuantStudio 5 instrument (Thermo Fisher) with the following temperature cycling: 95 °C for 10 min followed by 45 cycles consisting of 95 °C for 15 s and 60 °C for 1 min. Results were analysed using the QuantStudio Design & Analysis software (Thermo Fisher). Formula 2^−ΔΔCt^ was used to assess expression of the *MMACHC* and *PRDX1* genes, and *YWHAZ* was used as a reference (housekeeping) gene. Three independent experiments were performed with two technical replicates, with a mean intra-assay variation of 1.4%. The values are expressed relative to C1 blood expression levels to enable comparison across plates.

Aberrant *PRDX1* transcript detection and relative quantification were performed in triplicate using PCR from 1 µl of cDNA in a 10 µl reaction volume containing 0.2 µM specific primers (Suppl. Table 1), 0.025 U HotFirePol (Solis Biodyne), 1x Buffer B1, 2.5 mM MgCl_2_, 0.2 mM dNTP, and 0.1x SYBR Green I (Thermo Fisher). The cycling conditions were as follows: initial denaturation at 95 °C for 15 min, 40 cycles consisting of 94 °C for 30s, 62 °C for 1 min, and 72 °C for 1 min, followed by 72 °C for 7 min. No Ct value outliers were detected using Grubbs’ test (GraphPad), and the mean intra-assay variation was 1.2%. Expression of the *PRDX1* aberrant transcript was normalised to the reference gene *TBP* using the 2^−ΔΔCt^ formula and was expressed relative to the levels detected in the patient’s blood RNA. Detected PCR products were checked for specificity using melting curve plots and visually on 2% agarose containing GelRed (Biotium). Results from four independent experiments were used to estimate the mean and standard deviation.

## Results

### Patient description

The proband is a 65-year-old woman from Slovakia. Since her forties, she has suffered from headaches, limb numbness, progressive retinal dystrophy, pancytopenia, and hypertension. She developed polyneuropathy at the age of 59. She is university-educated and currently still working part-time in her specialty. During metabolic investigations in her 45th year, high urine MMA levels (14 583 mmol/mol of creatinine; normal range < 3.6 mmol/mol of creatinine) and mildly increased total Hcy plasma levels (28 µmol/l; normal range < 15 µmol/l) were found, while the serum cobalamin levels were normal (296 ng/l; normal range 160–950 ng/l). The patient was a confirmed compound heterozygote for the *MTHFR* polymorphisms c.665C>T, p.(Ala222Val) and c.1286A>C, p.(Glu429Ala), both associated with mild hyperhomocysteinemia. As a result, a diagnosis of isolated methylmalonic aciduria was proposed. The patient then underwent a therapeutic diagnostic test with oral administration of 1 mg cyanocobalamin three times a day for 1 week. Consequently, the MMA levels decreased to 1 045 mmol/mol of creatinine, which confirmed the cobalamin-responsive form of methylmalonic aciduria. At the age of 63, she underwent further genetic investigation. Her 38-year-old healthy daughter was also available for genetic testing. Due to the genetic heterogeneity of methylmalonic aciduria, WES was performed with targeted analysis of the virtual panel of genes associated with cobalamin metabolism. After confirmation of the *epi-cblC* type (see the next section), the patient was transferred to hydroxocobalamin (OH-Cbl) treatment. A dose of 1 mg/day i.m. decreased urine MMA from 679 to 120 mmol/mol of creatinine (normal range < 2.5 mmol/mol of creatinine) after 3 days. Due to poor patient tolerance, the dose was modified to 1 mg p.o./day and 1 mg i.m. once a week with good results (summarized in Suppl. Table 2). Plasma Hcy levels remained moderately elevated despite cobalamin treatment. It might be due to the patient’s low tolerance to frequent OH-Cbl injections and to higher betaine doses. However, other studies also reported that decreased Hcy levels remain above normal values [17, 18].

### Genetic analysis – WES, Sanger, co-segregation

Initial analysis of the WES data revealed the presence of a known pathogenic variant *MMACHC*:c.271dupA, p.(Arg91Lysfs*14) in a heterozygous state. No second variant was detected in this gene; however, a rare stop retained variant of unknown significance, *PRDX1*:c.599G >A, p.(Ter200=), was found. This variant changes the TGA stop codon to TAA and is present in five heterozygotes from the European non-Finnish population in gnomAD v4.0 (MAF 0.000004246). *In silico* predictions suggest a low probability of an effect on splicing (SpliceAI score 0.02, Pangolin score 0.17 on donor gain). Nevertheless, lacking a stronger candidate, we decided to examine the possibility of digenic *MMACHC*+*PRDX1 epi-cblC* in the patient. To support this, we tested the patient’s daughter for the presence of these two variants. She was found to carry only the heterozygous *PRDX1*:c.599G >A, p.(Ter200=) variant, which confirmed the *trans* localization of the *MMACHC* and *PRDX1* variants in her mother.

### *PRDX1*aberrant transcript

**Fig. 1:**
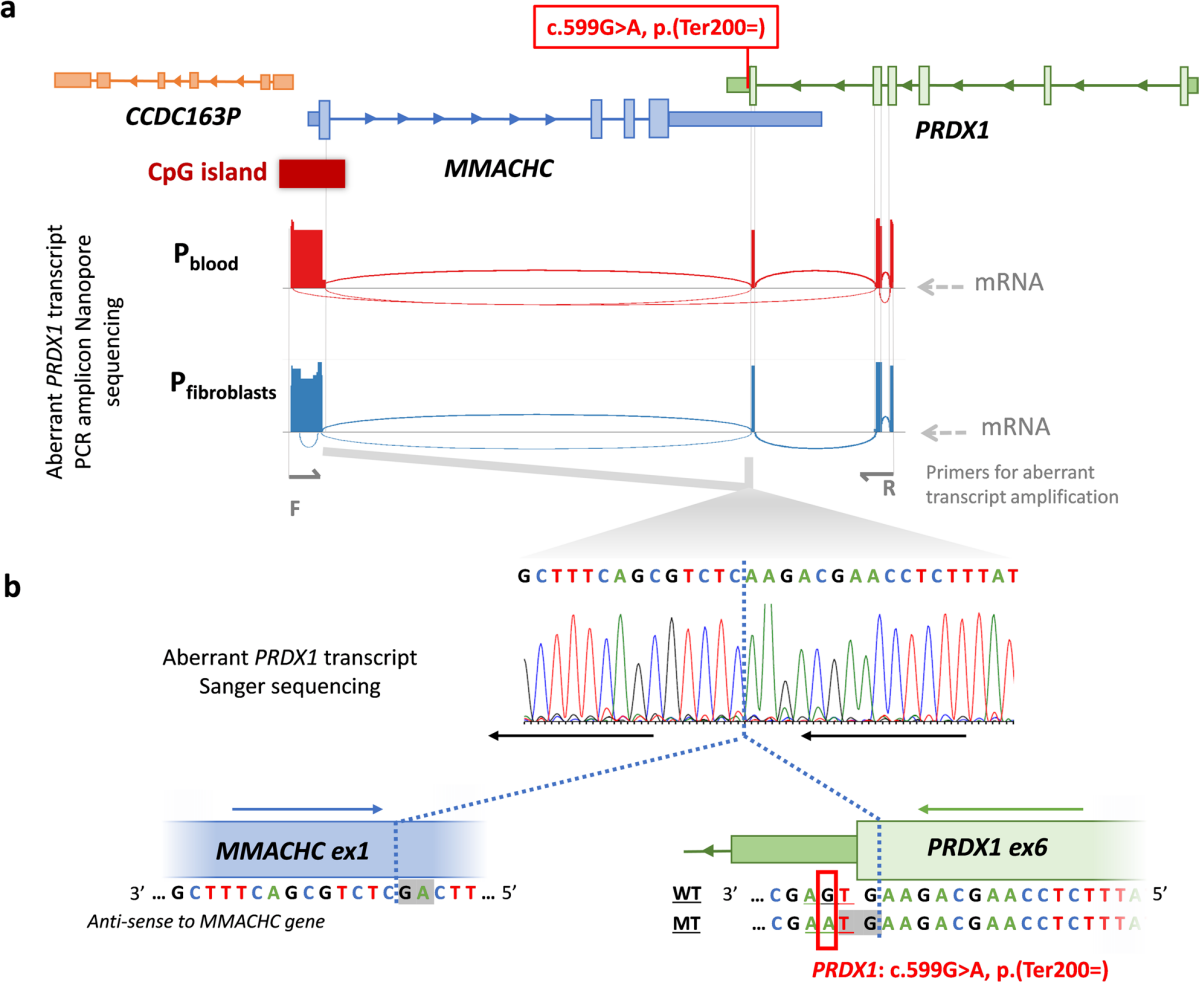
Aberrant *PRDX1* transcript. Schematic presentation of the *CCDC163P*, *MMACHC*, and *PRDX1* genes and a saschimi plot of transcripts detected in the patient’s blood and fibroblast RNA using primers specific for aberrant *PRDX1* transcript (**a**); Result of Sanger sequencing revealing consequences of the stop retained variant on splicing (**b**). TGA and TAA stop codons are underlined; the cryptic donor GT and acceptor AG sites used in aberrant splicing events are shaded grey

Previously described splice variants in the *PRDX1* gene associated with *epi-cblC* (c.515-1G>T and c.515- A>T) were shown to produce an aberrant transcript overlapping the *CCDC163P*/*MMACHC* bidirectional promoter [3, 4]. This overlap is proposed to induce methylation of the promoter and subsequently downregulate the *MMACHC* gene expression. Assuming the presence of a similar aberrant transcript in our patient, we used previously published primers [3] for amplification and Sanger sequencing of the aberrant transcript. Indeed, we confirmed its presence in the RNA from both blood and fibroblasts of the proband. Sanger and Nanopore sequencing revealed multiple alternatively spliced *PRDX1* transcripts spanning into the *MMACHC* first exon and promoter (Fig. 1a). It revealed that the *PRDX1*:c.599G>A, p.(Ter200=) variant in the stop codon resulted in utilization of a cryptic donor splice-site at the very end of the last *PRDX1* exon (Fig. 1b). Skipping of the whole *PRDX1* exon 6 was detected in a minority of transcripts in the patient’s blood RNA (Fig. 1a). It is worth noting that this approach can only reveal transcripts detectable by the used primers, and the presence of additional splice products cannot be ruled out.

### CCDC163P/MMACHC bidirectional promoter methylation

**Fig. 2:**
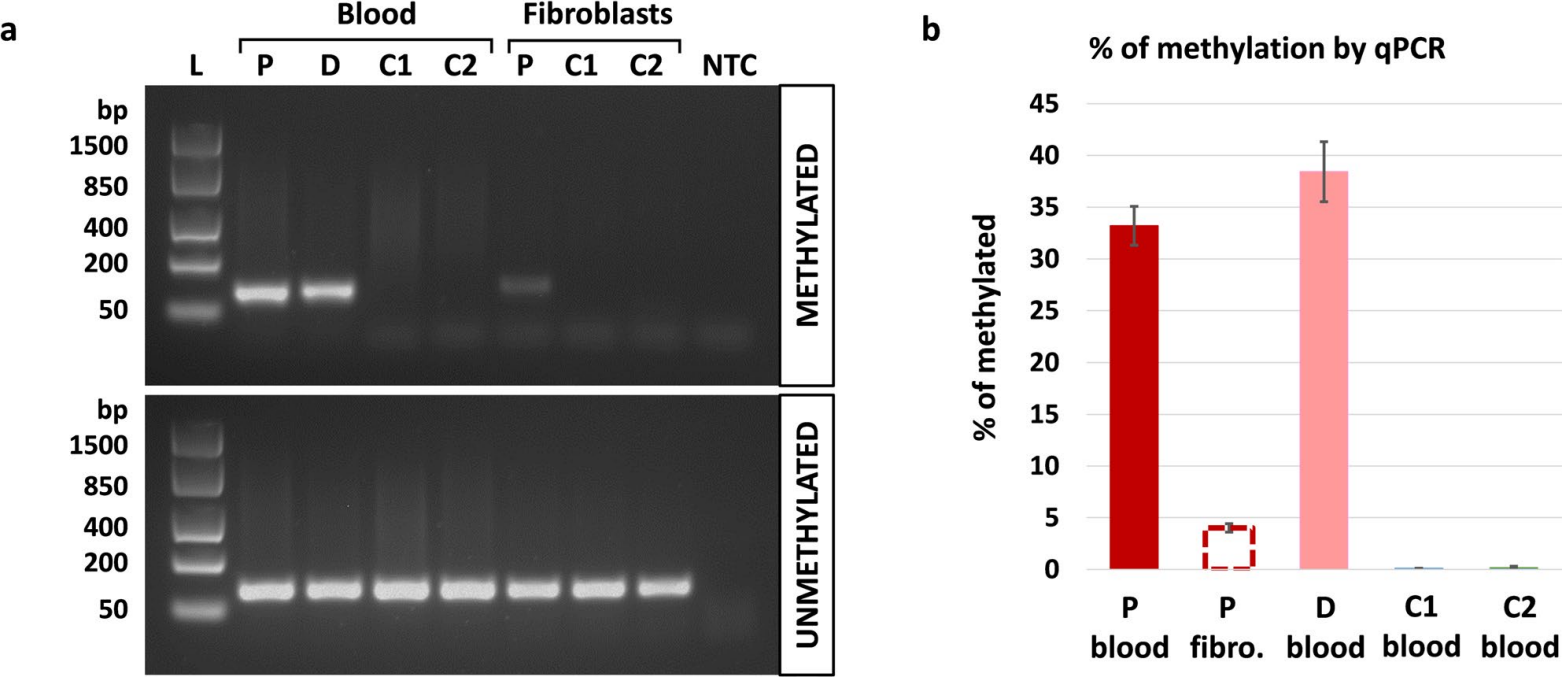
Methyl-specific PCR. BS-treated DNA amplified with primers specific for the unmethylated or methylated sequence of the *MMACHC* promoter (**a**); qPCR quantification of the methylated sequence (**b**). C1, C2 – control 1, control 2, D - daughter, Fibro. – fibroblasts, L – ladder, P - patient, NTC – no template control

As already mentioned, the production of the aberrant *PRDX1* transcript is linked to the methylation of the *CCDC163P*/*MMACHC* promoter. We assessed the promoter methylation status using methylation-specific PCR with BS-treated DNA, as described in [5]. The results confirmed the presence of both unmethylated and methylated *MMACHC* promoter sequences in DNA from the patient’s blood and fibroblasts, as well as in the blood DNA of her daughter, the *PRDX1* variant carrier (Fig. 2a). In healthy controls, only the unmethylated sequence was detected. This result was confirmed by qPCR with the same unmethylated and methylated sequence-specific primers (Fig. 2b). Interestingly, both methods showed a considerably lower methylation level in the patient fibroblasts compared to the blood DNA (Fig. 2).

**Fig. 3:**
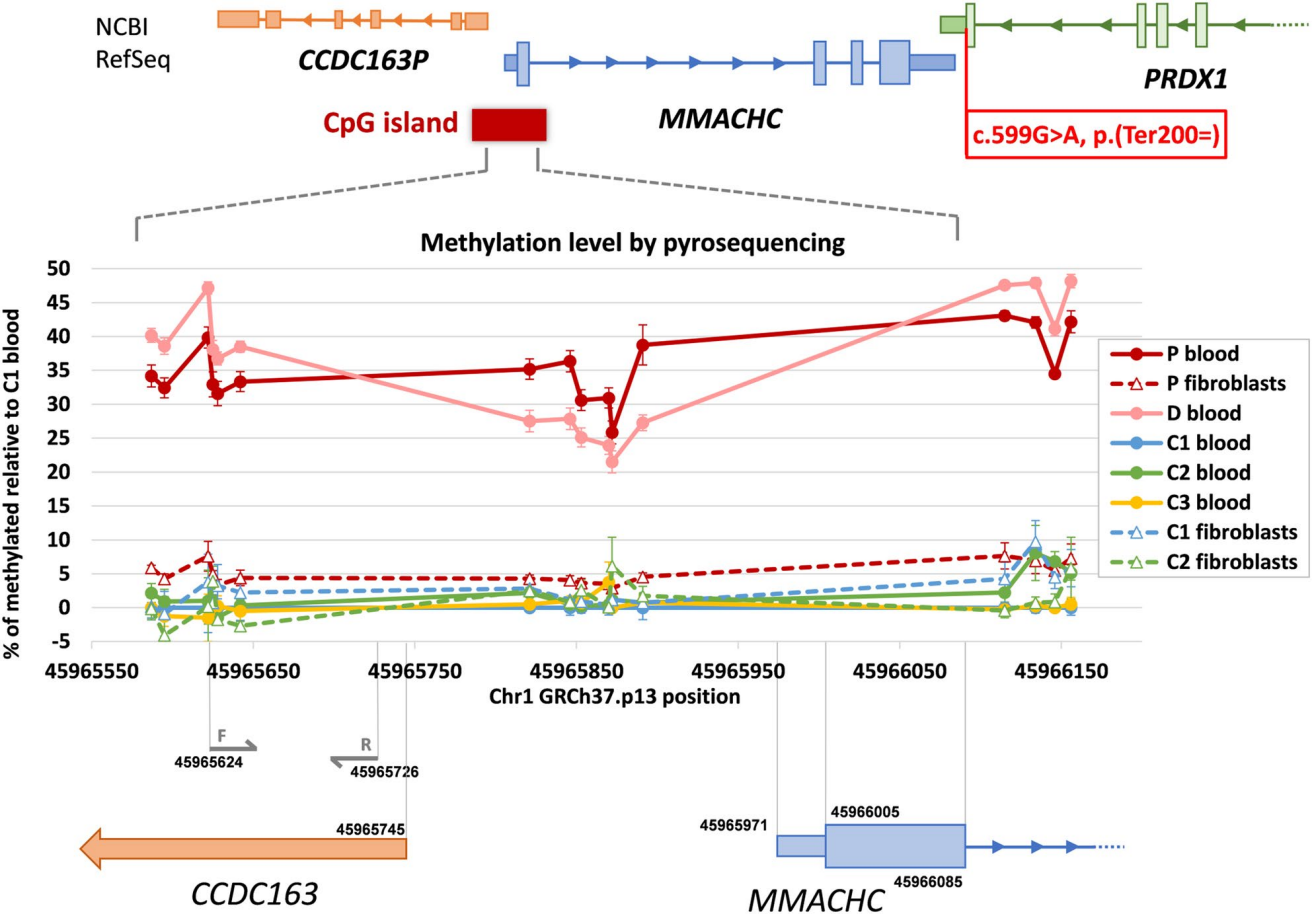
Quantification of methylation by pyrosequencing was done in three selected regions of the *MMACHC* promoter in BS-treated blood DNA of the patient (P), her daughter (D) and three healthy controls (C1, C2, C3), as well as in the BS-treated DNA from patient’s skin fibroblasts and fibroblasts of two healthy controls (C1, C2). The values were normalized to the C1 blood DNA control. The error bars represent the standard error of the mean. Primers F and R (grey) show the location of the target sequence for the methyl-specific PCR from Fig. 2

To determine whether the lower methylation level detected by PCR methods in the proband’s fibroblasts is really due to lower methylation of the *MMACHC* promoter or due to some unknown PCR-related issue, we decided to assess the methylation of multiple CpG positions in the *MMACHC* promoter region using pyrosequencing. We assessed methylation levels in three regions within the CpG island of the *CCDC163P*/*MMACHC* bidirectional promoter, covering 16 of the 33 CpG sites in this promoter. The precise locations of the covered CpG sites and the corresponding methylation levels are summarized in the Suppl. Table 3. We tested the DNA of the proband, her daughter, and three healthy controls, using DNA from both blood and fibroblasts where available (Fig. 3). After abstracting the background (C1 healthy blood DNA control values), the average methylation levels at these 16 CpG sites reached 35% and 36% in the DNA from the proband’s and her daughter’s blood, respectively. The methylation in the patient’s fibroblasts was confirmed to be low, 5% on average, approaching the lower limit of the method’s sensitivity. However, since the values for healthy controls ranged from 0 to 2.6% and the result is consistent with the values obtained by quantitative methyl-specific PCR (Fig. 2b), we are confident that the fibroblast DNA has a low level of methylation.

### MMACHC and PRDX1 expression in blood and fibroblasts

**Fig. 4:**
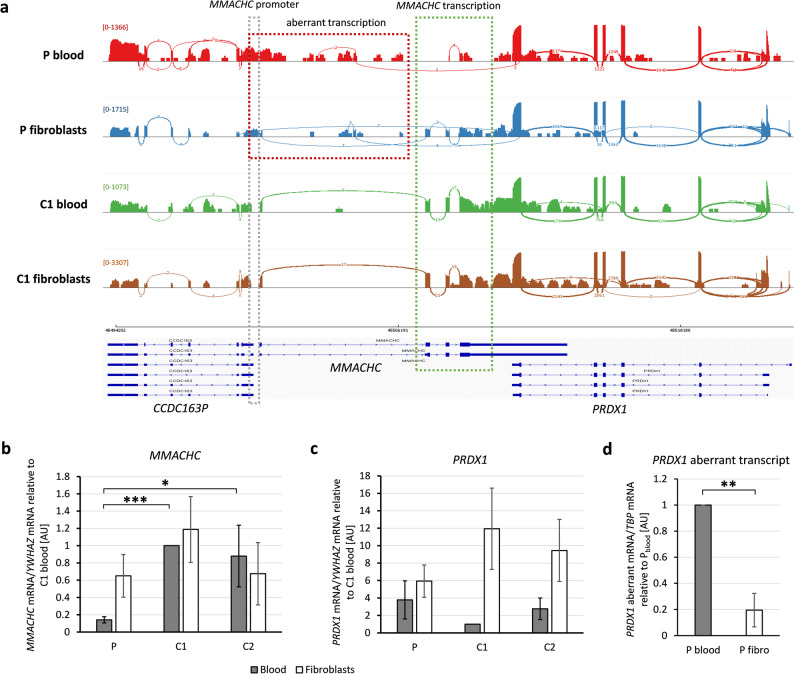
Expression of *MMACHC*, *PRDX1*, and aberrant *PRDX1* transcript. A saschimi plot generated from mRNA-seq data from blood or fibroblasts from the patient (P) and one of the control individuals (C1) shows multiple reads overlapping the *MMACHC* promoter and intron 1 sequence, as well as decreased *MMACHC* expression in the patient’s blood. The coverage on the y-axis is plotted on a log scale (**a**). The qPCR results showing *MMACHC* (**b**) and *PRDX1* (**c**) gene expression in blood and fibroblasts of the proband (P) and two healthy controls (C1 and C2). Expression was normalised to the *YWHAZ* reference gene and is expressed relative to C1 blood expression levels. Panel (**d**) shows the qPCR results for expression of *PRDX1* aberrant transcript overlapping the *CCDC163P*/*MMACHC* promoter in the patient’s blood and fibroblasts, normalised to *TBP* and expressed as relative to the blood expression levels

As the *MMACHC* promoter methylation levels differed in the patient’s blood and fibroblasts, expression from this allele should also be different. One allele harboring the frameshift *MMACHC* variant should be degraded in the process of nonsense-mediated decay in both tissues, and expression of the other one should correlate negatively with the detected level of promoter methylation. Indeed, mRNAseq data confirmed the almost complete absence of *MMACHC* transcripts in blood, consistent with suspected hemimethylation, whereas *MMACHC* transcripts were present in RNA from fibroblasts, where only a low level of methylation was detected (Fig. 4a). These results were confirmed by qPCR, which showed decreased *MMACHC* expression in the patient’s blood, while expression in fibroblasts was comparable to that of the controls (Fig. 4b).

It has previously been shown that the methylation level of the *CCDC163P*/*MMACHC* bidirectional promoter correlates with the expression of the aberrant *PRDX1* transcript [3]. To investigate whether the low methylation level in the patient’s fibroblasts could be explained by differences in *PRDX1* gene expression, we performed qPCR to quantify *PRDX1* gene expression in the patient’s blood and fibroblasts. However, we did not observe lower *PRDX1* gene expression level in the fibroblasts (Fig. 4c).

From previous mRNAseq studies, it is evident that the aberrant transcripts arising from the *PRDX1*:c.515-1G>T or c.515- A>T variants made only a minor portion of all processed *PRDX1* transcripts [3, 19]. Inspection of the mRNAseq data of our patient with the *PRDX1*:c.599G>A, p.(Ter200=) variant showed that the majority of the reads overlapping the *PRDX1* stop codon that came from the mutant c.599 A allele were processed correctly. Only 0.86% and 0.59% of these reads were spliced using the cryptic donor splice site in blood and fibroblasts, respectively. Degradation or a possible missing poly-A tail, which was used to enrich the mRNA library during RNAseq library preparation, could contribute to the low number of reads showing aberrant splicing. However, the reads from the correctly processed c.599 A mutant allele accounted for 38% and 44% of all reads at this position (coverage > 1200x and > 1500x) in the patient’s blood and fibroblast RNA, respectively. This indicates that the cryptic donor splice site was indeed activated in only a minority of *PRDX1* transcripts, and likely less frequently in fibroblasts. To confirm this, we quantified the expression of the aberrant transcript in our patient using qPCR with newly designed primers detecting the transcripts spanning the *PRDX1* exon 6 and the *CCD163P*/*MMACHC* promoter (Suppl. Table 1). A significantly lower level of aberrant transcript was observed in the patient’s fibroblasts compared to the blood (Fig. 4d), which could, finally, explain the lower methylation in the *MMACHC* promoter found in the patient’s fibroblasts.

## Discussion

In this study, we present a new case of *epi-cblC* methylmalonic aciduria due to digenic variants – the recurrent *MMACHC*:c.271dupA, p.(Arg91Lysfs*14) pathogenic variant and the newly described *PRDX1*:c.599G>A, p.(Ter200=) variant. The patient had unusually late-onset *epi-cblC*. To date, only three *epi-cblC* patients with later onset have been reported, and all carried the *PRDX1*:c.515-1G>T variant *in trans* with a missense variant (Suppl. Table 4). The first was the *MMACHC*:c.158T>C, p.(Leu53Pro) variant in patient WG-4152 in Gueant et al. [3], with onset at 59 years. Patient 10 in Cavicchi et al. [4] with onset in adulthood, reported also as MG-002151 in [3], carried the *MMACHC*:c.617G>A, p.(Arg206Gln) variant. The last variant, *MMACHC*:c.482G>A, p.(Arg161Gln) was reported by Pollini et al. [20] in a patient with presentation at 14 years. Pollini et al. did not assess the methylation level in their patients’ DNA; however, patient WG-4152 [3] had confirmed hemimethylation in blood and fibroblasts, and patient 10 [4] had confirmed hemimethylation in blood, which should lead to the monoallelic expression of the allele carrying the missense variant. It is therefore probable that, at least in these two cases, the missense variants conferred some residual activity. Indeed, the *MMACHC*:c.617G>A, p.(Arg206Gln) variant has been suggested to be associated with milder outcomes of *cblC* [4]. The p.(Arg161Gln) variant has been associated with late-onset presentation and milder phenotype [21, 22]. Froese et al. have demonstrated that the MMACHC protein with this mutation remains functional, albeit with impaired cobalamin-induced stabilization and increased half-maximal effective ligand concentration [23]. Patients carrying the *MMACHC*:c.158T>C, p.(Leu53Pro) variant also had mild clinical and biochemical phenotypes and responded well to hydroxocobalamin monotherapy [18].

Our patient had the epimutation in combination with the most frequent *MMACHC*:c.271dupA, p.(Arg91Lysfs*14) pathogenic variant. The mRNAseq data of our patient confirms that there is almost no expression from either of the *MMACHC* alleles in blood, and no expression was detected from the allele with the *MMACHC*:c.271dupA variant in the patient’s fibroblasts. This is in agreement with presumed nonsense-mediated decay of the transcript, where the frameshift mutation introduces a premature termination codon. This frameshift variant has a severe impact and is associated with early-onset and severe disease if homozygous [24]. In agreement with this, all patients in previous studies carrying an epi-mutation *in trans* with the *MMACHC*:c.271dupA variant had early onset of the disease. In contrast, our patient had the onset of the disease as late as her forties. The later onset should therefore be due to the *PRDX1* variant.

We showed that the *PRDX1*:c.599G>A, p.(Ter200=) variant, though only changing one stop codon for another one, induces changes in splicing, despite benign *in silico* predictions. To our knowledge, this is one of the very scarce, if not unique, examples of a stop retained variant with confirmed pathogenicity. Another non-coding variant in this region has previously been reported in an *epi-cblC* patient, *PRDX1*:c.*2C > T [5]. Its precise effect on splicing was not studied, but we can expect it to result in aberrant transcript production, too, as it causes the epimutation. Nevertheless, the pathogenicity of this change in splicing is due to the specific mechanism involving methylation of the opposite DNA strand in specifically organised reverse (R1)/forward (F2)/reverse (R3) trios of genes [6] and cannot be applied universally. Our case demonstrates that a relatively low level of aberrantly spliced *PRDX1* transcripts can lead to *MMACHC* promoter methylation, possibly due to the inherently high expression of *PRDX1* in the affected tissues. Therefore, the threshold may differ in cases of other genes, depending on their overall level of expression. This knowledge can have further implications for the future classification of *PRDX1* variants or for the training of tools predicting splice changes.

The production of aberrant *PRDX1* mRNA overlapping the *CCDC163P*/*MMACHC* bidirectional promoter presumably led to the epimutation we confirmed in the two *PRDX1*:c.599G>A, p.(Ter200=) variant carriers. The DNA from blood was hemimethylated in both the patient and her daughter; however, the level of methylation in the patient’s fibroblasts reached only ~ 5% level. In line with this, *MMACHC* expression was decreased in the patient’s blood but was preserved in the fibroblasts. The unusually late disease onset in our patient could therefore be due to lower methylation levels in tissues other than blood. Such a discrepancy in methylation levels of different tissues has not yet been described in *epi-cblC* patients. All published DNA samples from fibroblasts of subjects carrying the heterozygous epi-mutation due to *PRDX1*:c.515-1G>T were hemimethylated, and the *MMACHC* promoter of a patient with bi-allelic *PRDX1*:c.515-1G>T was fully methylated, with the complete silencing of *MMACHC* expression [4].

**Fig. 5:**
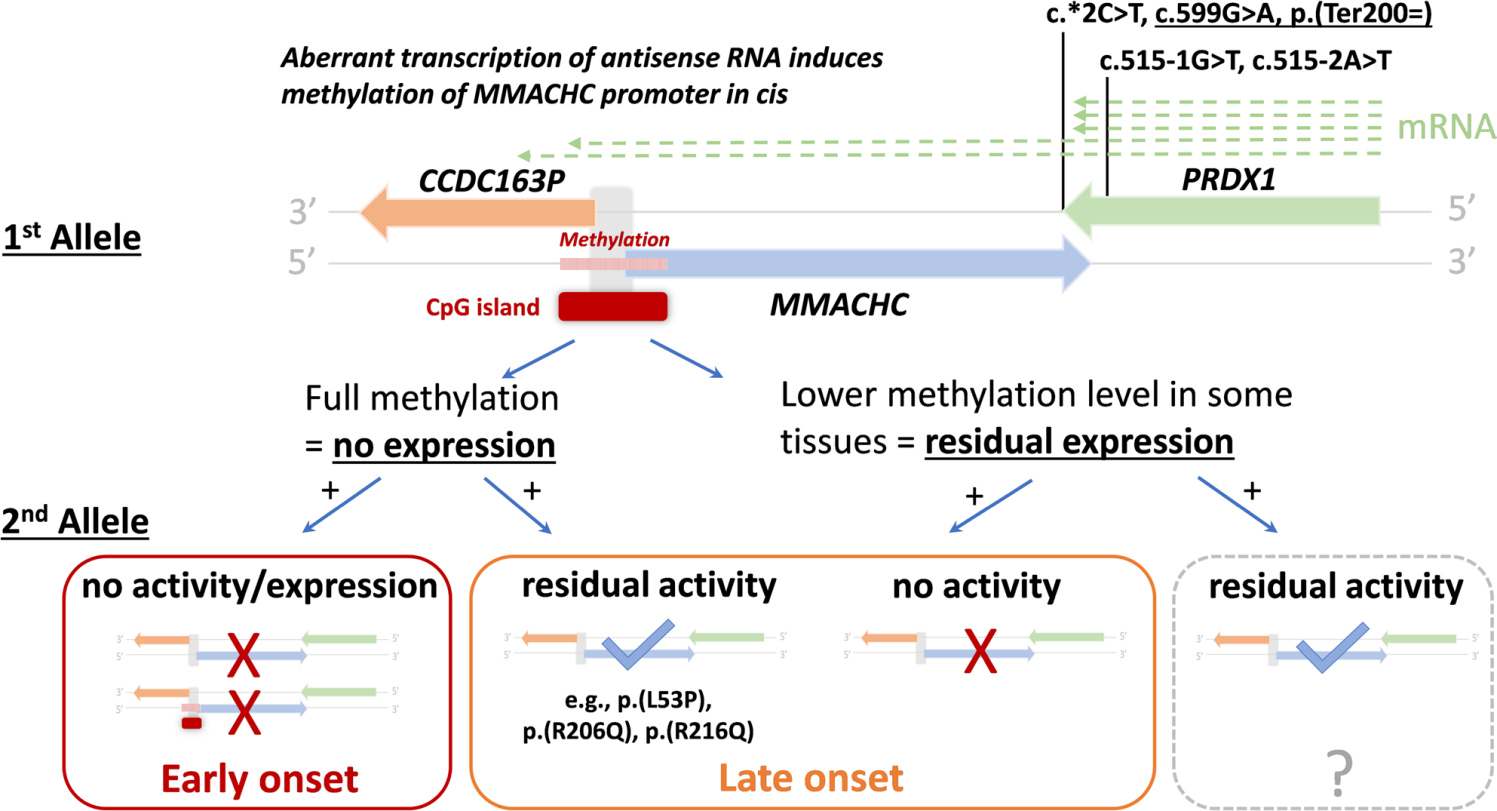
Suggested model for the development of late-onset *epi-cblC*. The scheme summarizes the *PRDX1* variants that cause aberrant transcription leading to methylation of the *MMACHC* promoter (the underlined variant is reported in this study). Methylation of the allele can be full, leading to hemimethylation in the case of a heterozygous epimutation, or to full methylation in the case of a bi-allelic epimutation. A complete lack of a functional product from the *MMACHC* gene leads to early-onset *epi-cblC*. Late-onset * epi-cblC* develops when this is combined with an *MMACHC* variant that retains residual activity, or when there is lower methylation in at least some tissues

Gueant et al. [3] showed that the methylation level of the *CCDC163P/MMACHC* promoter is dependent on the expression of the *PRDX1* gene. When they silenced *PRDX1* expression with siRNA, the methylation level of the promoter decreased, and expression of the *MMACHC* gene was restored. We therefore measured the expression of the *PRDX1* and *MMACHC* genes in RNA from blood and fibroblasts of the patient and the controls to determine whether *PRDX1* expression correlates with methylation. According to the GTEx portal data [25] (accessed January 2026, version V10), *PRDX1* expression is higher in the cultured fibroblasts (median transcripts per million (TPM) 470) than in blood (median TPM 52). In our experiment, we also observed a trend towards higher *PRDX1* mRNA levels in fibroblasts than in whole blood in the controls, and these levels were comparable in the patient (Fig. 4c). This means that the lower methylation in the patient fibroblasts is not due to differences in the overall *PRDX1* expression in these two tissues. However, the significantly lower expression of aberrant *PRDX1* transcripts in the patient fibroblasts compared to blood (Fig. 4d) could explain the lower methylation levels and preserved *MMACHC* expression in this tissue. Thus, the expression of the aberrant *PRDX1* transcript under the already low threshold leads to lower methylation, which might contribute to the variability in the severity of the *epi-cblC* outcome among individual patients (Fig. 5).

The mechanism by which the *PRDX1*:c.599G>A, p.(Ter200=) variant leads to a lower proportion of aberrant splicing events in the fibroblasts compared to blood can be related to tissue-specific differences in the splicing machinery or aberrant mRNA degradation pathways and remains to be elucidated in further studies, favourably including more individuals and tested tissues or in vitro studies. 

### Limitations 

The causative link between the antisense RNA and methylation is inferred from genetic association and prior studies rather than proven via functional experimentation in this work. The precise reason for the observed tissue-specific difference in methylation (blood vs. fibroblast) remains an open and interesting question for future research.

## Conclusion

In our study, we demonstrated a mix of multiple interesting molecular biology processes, which ultimately led to late-onset *epi-cblC* in the patient. We highlight the unusual outcome of a stop retained variant, which, despite influencing only a small proportion of transcripts, caused aberrant splicing that led to repression of expression in an adjacent gene through DNA methylation. Furthermore, we observed that aberrant splicing occurred at varying levels in blood and fibroblasts, which could explain the lower methylation of the *MMACHC* promoter in the patient’s fibroblasts and, eventually, explain the late onset of the disease in our patient.

## Supplementary Information

Below is the link to the electronic supplementary material.

Supplementary Material 1

Supplementary Material 2

## Data Availability

The datasets supporting the conclusions of this article are included within the article and its additional files.
