# Supplementary material for: Late-onset epi-cblC methylmalonic aciduria with tissue-variable MMACHC promoter methylation due to a stop retained PRDX1 variant
Source: Clin Epigenetics. 2026 Apr 19;18:141. doi: 10.1186/s13148-026-02135-8 (PMC13366942; doi:10.1186/s13148-026-02135-8)
Supplement: Supplementary file 1 — Supplementary Material 1 [file 13148_2026_2135_MOESM1_ESM.pdf]

Supplementary Table 1: List of primers used in the study.

	5' to 3' sequence	Modification	Target	Amplicon length	Reference
**Sanger sequencing**					
MMACHC-2F	TGCATCACATAGCGTCAGTG		gDNA	467 bp	Zhang et al., 2021
MMACHC-2R	AGCCTGGCTTTAGGGTATCA		gDNA		Zhang et al., 2021
PRDX1-6F	TCCATAGGAGAATGGTGGTGC		gDNA	283 bp	Zhang et al., 2021*
PRDX1-6R	GCCTGCCTTGTAAGACACCACA		gDNA		Zhang et al., 2021
**Methyl-specific MMACHC promoter PCR**					
MSP-F	TCGTCGGGTTTAAAATTTC		BS-treated gDNA	103 bp	Zhang et al., 2021
MSP-R	CCGATTATATTCGCAAAAAA		BS-treated gDNA		Zhang et al., 2021
UM-F	GTTGTTGGGTTTAAAATTTT		BS-treated gDNA	103 bp	Zhang et al., 2021
UM-R	CCAATTATATTCACAAAAAAC		BS-treated gDNA		Zhang et al., 2021
**Pyrosequencing primers**					
MMACHC_pyro_reg1_F	GGGGATTGAAGGTGTTAGAA		BS-treated gDNA		This study
MMACHC_pyro_reg1_R	CCTCCTTAAAAACAACCACAC	5'Biotin	BS-treated gDNA		This study
MMACHC_pyro_reg1_S	GTTGAGATTTTTTTAGAAGTAGT		BS-treated gDNA		This study
MMACHC_pyro_reg2_F	AGGAGGTGGAATTATTTTTTTTTAGAAGAG		BS-treated gDNA		This study
MMACHC_pyro_reg2_R	ACTCCATTTCCCAAATAAAAAAATAAATC	5'Biotin	BS-treated gDNA		This study
MMACHC_pyro_reg2_S	GGAAATTGAGTTTTTTTGAT		BS-treated gDNA		This study
MMACHC_pyro_reg3_F	AGGTTTATTTTTTTTAGGTTAGTTTAT		BS-treated gDNA		This study
MMACHC_pyro_reg3_R	AAAACCCAAAAAAATCAAAAACTCC	5'Biotin	BS-treated gDNA		This study
MMACHC_pyro_reg3_S	TTAGTTTATTTTTTTTGTTGTTTTA		BS-treated gDNA		This study
**Aberrant PRDX1 transcript Sanger sequencing**					
PRDX1_S	CATTCCTTTGGTATCAGACCCG		cDNA	variable	Gueant et al., 2018
CCDC163P_AS	AGCGTTGAGAAGCACATCCA		cDNA		
**Aberrant PRDX1 transcript quantification**					
qPCR_PRDX1_aberr_F	CAGTGATACCATCAAGCCTG		cDNA	140 bp	This study
qPCR_PRDX1_aberr_R	CGTAACGGCCCAATTGTC		cDNA		

** Named PRDX1-5F in Zhang et al., 2021*

Supplementary Table 2: Biochemical parameters of the patient related to epi-CblC disease progression monitoring.

Therapy			Age [yrs]	Metabolic findings					
Cobalamin		Folic acid dose		MMA urine (N:<2.5 mmol/mol creatinine)	MMA serum (N:< 0.4 µmol/l)	tHcy plasma (N: 5-15 µmol/l)	Methylcitrate (N: <0.1 mmol/mol creatinine*)*	Folic acid serum (N:4.5-20.7 nmol/l)	Cbl serum (N:160-950 ng/l)
Time period	Dose								
Before therapy during decompensation	-		45	14 583	n.a.	28	n.a.	31	296
CN-Cbl – 1-week therapeutic diagnostic test	3 x 1 mg p.o./day		45	4 670	n.a.	n.a.	n.a.	n.a.	n.a.
- long-term	1 mg i.m. once in 2 weeks	10 mg 3x/week	45-63	1 045	n.a.	57…54… 49…34…40 …38…54*	n.a.	41	421
- immediately before switch	1 mg i.m. once in 2 weeks	10 mg 3x/week	63	679.4	94.1	40.8	10	78.4	1 084
OH-Cbl – 3 days after switch to OH-Cbl	1 mg i.m./day	10 mg 3x/week	63	120	n.a.	n.a.	traces	n.a.	n.a.
- 6 weeks	1 mg i.m. once weekly + 1 mg p.o./day	10 mg 3x/week	63	112	14.8	26.2	1.1	n.a.	n.a.
- 6 months	1 mg i.m. once weekly + 1 mg p.o./day	10 mg 3x/week	64	42.4	4.1	28.2	1.8	n.a.	>1476
- 1 year	1 mg i.m. once weekly + 1 mg p.o./day	10 mg 3x/week	64	n.a	7.1	28	n.a	96	11 419
- 2 years	1 mg i.m. once weekly	10 mg 3x/week	65	158.6	n.a	29.6	1.6	>45.4	>1476

*Abbreviations: Cbl – cobalamin, CN-Cbl – cyanocobalamine, i.m. – intramuscular, MMA – methylmalonic acid, N – normal range, OH-Cbl - hydroxocobalamine, p.o. – per oral, tHcy – total homocysteine. * Values in order of measurements during the given time period.*

Supplementary Table 3: Percentage of % methylated cytosines at given positions in the MMACHC promoter determined by pyrosequencing. The regions 1 to 3 correspond to the three amplicons amplified and sequenced using primers listed in Supplementary Table 1.

	Region 1						Region 2						Region 3				
**GRCh37 position**	**45965587**	**45965595**	**45965622**	**45965625**	**45965628**	**45965642**	**45965821**	**45965846**	**45965853**	**45965870**	**45965872**	**45965891**	**45966115**	**45966134**	**45966146**	**45966156**	
**cg ID**	**-**	**cg16521245**	**-**	**cg13848568**	**-**	**-**	**-**	**cg27393325**	**-**	**cg22536808**	**-**	**-**	**cg15896098**	**-**	**-**	**-**	
**% of methylated**																	**AVERAGE**
P blood	40.7	43.2	55.8	38.2	35.8	40.3	37.7	42.8	34.3	38.2	28.3	46.0	44.8	45.3	37.0	45.7	40.6
P fibroblasts	12.4	15.0	23.6	10.2	7.6	11.4	6.8	10.6	7.4	10.8	5.4	11.8	9.4	10.2	8.0	10.8	10.7
D blood	46.7	49.3	63.2	43.3	41.0	45.5	30.0	34.3	28.8	31.2	24.0	34.5	49.3	51.2	43.7	51.7	41.1
C1 blood	6.5	10.8	16.0	5.3	4.3	7.0	2.5	6.5	3.8	7.3	2.5	7.3	1.8	3.3	2.5	3.5	5.8
C2 blood	8.7	11.7	17.0	5.3	2.8	7.3	4.7	7.0	4.0	7.7	3.7	8.0	4.0	11.3	9.3	8.7	7.5
C3 blood	6.5	9.5	14.5	5.0	4.5	6.5	3.0	7.5	4.5	11.0	2.5	8.0	1.5	3.5	2.5	4.0	6.0
C1 fibroblasts	6.7	9.8	19.8	6.0	7.5	9.3	5.3	7.7	4.7	7.7	3.7	8.0	6.0	13.0	7.0	9.3	8.1
C2 fibroblasts	6.3	6.7	16.2	9.2	2.5	4.3	5.0	7.3	6.3	7.3	8.7	9.0	1.3	4.0	3.3	9.3	6.5
**SEM**																	
P blood	1.6	1.5	1.6	1.8	1.8	1.5	1.5	1.6	1.5	1.5	1.7	2.9	0.7	0.8	0.4	1.6	
P fibroblasts	0.4	0.7	2.2	0.5	0.8	1.2	0.5	0.6	0.7	0.3	0.5	0.6	1.9	1.9	1.7	2.1	
D blood	1.0	1.2	0.9	1.3	1.0	0.8	1.6	1.6	1.4	1.3	1.6	1.1	0.6	0.8	1.1	1.0	
C1 blood	0.9	0.8	1.8	1.0	0.6	0.9	0.3	0.6	0.4	0.5	0.3	0.9	0.2	0.4	0.3	0.6	
C2 blood	1.4	1.8	3.4	0.8	0.5	0.8	0.3	0.0	0.0	0.3	0.3	0.5	1.4	4.1	1.4	1.1	
C3 blood	1.1	1.1	2.5	1.4	1.1	1.8	0.0	0.4	0.4	2.1	0.4	0.7	0.4	0.4	0.4	0.7	
C1 fibroblasts	1.7	3.3	4.1	0.3	3.1	2.2	0.5	0.3	0.3	0.3	0.3	0.5	2.4	3.1	1.7	2.8	
C2 fibroblasts	1.6	2.2	5.4	2.2	0.3	0.6	0.5	0.3	1.5	0.3	4.2	1.4	1.1	0.8	1.2	4.6	
**% of methylated normalised to C1 blood**																	**AVERAGE**
P blood	34.2	32.4	39.8	32.9	31.6	33.3	35.2	36.3	30.6	30.9	25.8	38.8	43.1	42.1	34.5	42.2	35.2
P fibroblasts	5.9	4.3	7.6	5.0	3.4	4.4	4.3	4.1	3.7	3.6	2.9	4.6	7.7	7.0	5.5	7.3	5.1
D blood	40.2	38.6	47.2	38.1	36.8	38.5	27.5	27.8	25.1	23.9	21.5	27.3	47.6	47.9	41.2	48.2	36.1
C1 blood	0.0	0.0	0.0	0.0	0.0	0.0	0.0	0.0	0.0	0.0	0.0	0.0	0.0	0.0	0.0	0.0	0.0
C2 blood	2.2	0.9	1.0	0.1	-1.4	0.3	2.2	0.5	0.3	0.4	1.2	0.8	2.3	8.1	6.8	5.2	1.9
C3 blood	0.0	-1.3	-1.5	-0.3	0.3	-0.5	0.5	1.0	0.8	3.8	0.0	0.8	-0.3	0.3	0.0	0.5	0.3
C1 fibroblasts	0.2	-0.9	3.8	0.8	3.3	2.3	2.8	1.2	0.9	0.4	1.2	0.8	4.3	9.8	4.5	5.8	2.6
C2 fibroblasts	-0.2	-4.1	0.2	3.9	-1.8	-2.7	2.5	0.8	2.6	0.1	6.2	1.8	-0.4	0.8	0.8	5.8	1.0

Abbreviations: C1, C2, C3 - healthy controls; D - patient's daughter; P - patient

Supplementary Table 4: Characteristics of the patients with late-onset *epi-CblC* reported in the literature and metabolic findings at biochemical diagnosis.

Patient	Genotype		MMACHC promoter methylation	Age of onset	Age of dx	MMA urine	Hcy plasma	Clinical Features	
	Allele 1 (*MMACHC*, NM_015506)	Allele 2 (*PRDX1*, NM_181697)							
Proband, P	*MMACHC*:c.271dupA, p.(Arg91Lysfs*14)	*PRDX1*:c.599G>A, p.(Ter200=)	Blood – hemimethylation; Fibroblasts - ~5 %	>40 years	63 years	14 583 mmol/mol creatinine (N: <2.5 mmol/mol creatinine)	28 µmol/l (N: 5-15 µmol/l)	headaches, limb numbness, progressive retinal dystrophy, pancytopenia, hypertension, polyneuropathy	**This study**
WG-4152	*MMACHC*:c.158T>C, (p.Leu53Pro)	*PRDX1*:c.515-1G>T	Fibroblasts - hemimethylation; blood - hemimethylation	59 years	59 years	9.0 µmol/l	99 µmol/l	oral thrush and vomiting	Gueant et al., 2018 [1]
Patient	*MMACHC*:c.482G>A, p.(Arg161Gln)	*PRDX1*:c.515-1G>T	N/A	14 years	16 years	1 075 mmol/mol creatinine (N: 0.4–23 mmol/mol creatinine)	162 µmol/l (N: 4.7–11.3 µmol/l)	decline in school performance and a loss of strength, sudden visual loss, spastic paraparesis, loss of ambulation at 16 years of age, dystonia, hypertrophic cardiomyopathy, and proteinuria	Pollini et al., 2020 [2]
Pt 10	*MMACHC*:c.617G>A, p.(Arg206Gln)	*PRDX1*:c.515-1G>T	Blood - hemimethylation	<63 years, likely 3rd decade	75 years	2 706 mmol/mol creatinine (N: <2 mmol/mol creatinine)	357 µmol/l (N: <15 µmol/l)	Limb and abdomen pains (3-10 y); acute nephritis and anaemia (29 y); membranoproliferative glomerulonephritis and xerophthalmia secondary to SLE (30 y); hypertension (38 y); dysphonia (44 y); paroxysmal atrial fibrillation (45 y); deep venous thrombosis (49 y); sensorimotor polyneuropathy and antiphospholipid syndrome (50 y); pulmonary thromboembolism and homocystinuria (53 y); several transient ischemic attacks (56-60 y); distal tremors (60 y); subarachnoid haemorrhage (61 y); thrombosis of left renal artery (62 y); APCA positivity (63 y), Chronic gastritis and dolichocolon with colic melanosis (64 y); asthenia and severe back and limb pains (65 y); xerophthalmia (68 y); second episode of paroxysmal atrial fibrillation (70 y); deterioration of renal function, diffuse hypotonia, worsening of polyneuropathy with motor coordination deficit and white matter lesions at MRI compatible with gliosis and vasculitis (71 y)	Cavicchi et al., 2021 [3]

Abbreviations: APCA - anti-parietal cell antibodies, dx – diagnosis, Hcy – homocysteine, MMA – methylmalonic acid, N – normal range, SLE - systemic lupus erythematosus.

References

1. Gueant JL, Chery C, Oussalah A, Nadaf J, Coelho D, Josse T, Flayac J, Robert A, Koscinski I, Gastin I *et al*: **APRDX1 mutant allele causes a MMACHC secondary epimutation in cblC patients**. *Nat Commun* 2018, **9**(1):67.
2. Pollini L, Tolve M, Nardecchia F, Galosi S, Carducci C, di Carlo E, Carducci C, Leuzzi V: **Multiple sclerosis and intracellular cobalamin defect (MMACHC/PRDX1) comorbidity in a young male**. *Mol Genet Metab Rep* 2020, **22**:100560.
3. Cavicchi C, Oussalah A, Falliano S, Ferri L, Gozzini A, Gasperini S, Motta S, Rigoldi M, Parenti G, Tummolo A *et al*: **PRDX1 gene-related epi-cblC disease is a common type of inborn error of cobalamin metabolism with mono- or bi-allelic MMACHC epimutations**. *Clin Epigenetics* 2021, **13**(1):137.
