# Supplementary figures and images for: Late-onset epi-cblC methylmalonic aciduria with tissue-variable MMACHC promoter methylation due to a stop retained PRDX1 variant
Source: Clin Epigenetics. 2026 Apr 19;18:141. doi: 10.1186/s13148-026-02135-8 (PMC13366942; doi:10.1186/s13148-026-02135-8)

Supplementary file: Uncropped gel image used in Figure 2.


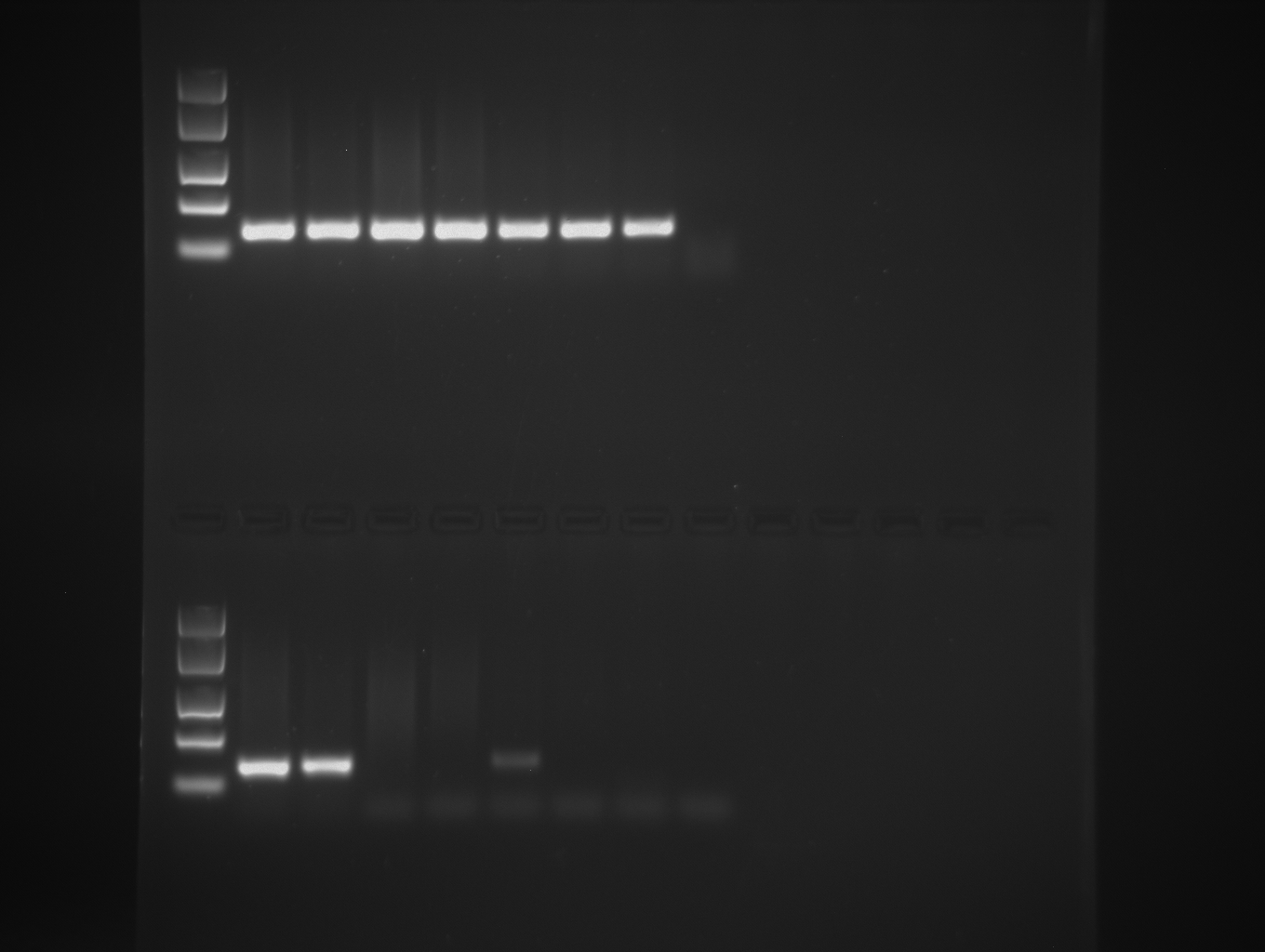

Supplement: Supplementary file 2 — Supplementary Material 2 [file 13148_2026_2135_MOESM2_ESM.docx]
